# Concerns regarding the study “Comparing the effects of a commercial and a prototype vitrification medium on meiotic spindle morphology and survival rate of mouse oocytes”

**DOI:** 10.5935/1518-0557.20220038

**Published:** 2022

**Authors:** Carlos Gilberto Almodin

**Affiliations:** 1 Materbaby - Human Reproduction and Genetics Clinic, Maringá, Brazil

Dear Editor,

Ref**: “Comparing the effects of a commercial and a prototype vitrification medium on meiotic spindle morphology and survival rate of mouse oocytes”.** Published on JBRA Assist Reprod. 2022;26:500-7 (Viana *et al*., 2022).

All research is very welcome to our community. But the researchers should follow the science rules, only this way someone can contribute to all the word. When we intend to get a paper from a research, it means we get the expertise from the other author and compare the technique and results with our research. Unfortunately, I read the paper above comparing it with my research. In this paper it is very clear that the authors have no experience with the technique I created and got several mistakes that compromised the research they performed.

To be honest, I am very concerned about the quality of this paper. The authors compared the vitrification medium that I create in 2006 with a homemade medium. My concerns are:

1. I have never suggested vitrification of 4 oocyte per stick. This pushes the result down.
2. The author of the paper has no experience with Ingamed medium. They have not used the correct protocol.
3. It is not possible to warm up properly the oocytes using 250 µl of medium. We use from 0,5ml to 1 ml. I can’t believe that they got the same result using this amount of medium to warm up the oocytes.
4. As a reference, there is the paper we have published on Human Reproduction (Almodin *et al*., 2010). In this work, we have described the protocol in details. It should be used to reproduce the method and get the same results. The author has used my paper as reference, but has not followed the protocol that I described.
5. Also, I understand the paper has to mention the material and it´s source of what was used in the experiment. This allows others to reproduce it. However, the author used 2 amino acids and didn’t show which one. This is a ghost ammoniac.
